# Supplementary material for: Comparative Risk of Complications Following Intestinal Surgery After Infliximab, Vedolizumab, or Ustekinumab Treatment: Systematic Review & Meta-Analysis
Source: Pharmaceuticals (Basel). 2025 Sep 29;18(10):1466. doi: 10.3390/ph18101466 (PMC12567440; doi:10.3390/ph18101466)
Supplement: Supplementary file 1 [file pharmaceuticals-18-01466-s001.zip › pharmaceuticals-3854303-supplementary.pdf]

<u>Supplementary Materials</u>

Table S1. Baseline Characteristics of Included Studies; Patients' Details

	Study	***Male, n (%)***	***Age, years (mean ± SD)***	***BMI***
**1**	**Tang** Shasha et al; (2020)	64.4%	36.3	18.9
**2**	El-**Hussuna** Alaa et al; (2018)	46%	42.5	23.5
**3**	**Ward** S. T et al; (2017)	58%	<18y.o=66 (8.76%)<br>18-30y.o=253 (33.6%)<br>31-50y.o=255 (33.8)<br>51-70y.o=155 (20%)<br>>70y.o=24 (3.19%)	
**4**	**Zittan** Eran et al; (2016)	58%	35	24.75
**5**	p. **Myrelid** et al; (2014)	48%	36.1	
**6**	**Uchino** Motoi et al; (2013)	70%	37	19
**7**	**Uchino** Motoi et al; (2013)	63%	43	19
**8**	**Krane** Mukta K et al; (2013)	53%	37	25
**9**	**Waterman** Matti et al; (2013)	43%	21	23
**10**	**Eshuis** Emma J et al; (2013)	57%	35	23
**11**	**Norgard** B M et al; (2013)	42.6%	37.3	
**12**	**Kasparek** M. S et al; (2012)	47%	37	21.7
**13**	**Norgard** B M et al; (2012)	77%	39.6	
**14**	**Bregnbak** D. et al; (2012)	49%	35.7	
**15**	**Regueiro** Miguel et al; (2011)	34.3%	45	
**16**	**Gainsbury** M. L et al; (2011)	40.1%	39.1	27.3
**17**	**Coquet-Reinier** B. et al; (2010)	53.8%	33.45	21.05
**18**	**Kunitake** Hiroko et al; (2008)	49.2%	36.95	
**19**	**Schluender** Stefanie J et al; (2007)	42%	33	
**20**	**Selvasekar** C. R et al (2007)	54.5%	33.5	24.5
**21**	**Poylin** Vitaliy Y et al; (2022)	47.4%	40.55	24.72
**22**	**Kim** Jeong Yeon et al; (2020)	50.5%	25	22.6
**23**	**Novello** M et al; (2020)			25.3
**24**	**Park** K T et al; (2018)	57.5%	38.4	
**25**	**Kotze** P.G. et al; (2018)	28%	53	27.7
**26**	**Lightner** Amy L et al; (2018)	46%	33	
**27**	**Lightner** A L et al; (2018)	26.1%	26.3	23.2
**28**	**Lightner** Amy L et al; (2017)	54.95%	43	25.55
**29**	**Ferrante** Marc et al; (2017)	51.5%	38.55	
**30**	**Yamada** Akihiro et al; (2017)	48.2%	38.76	
**31**	**Shah** Ravi S et al; (2021)	45.5%	36	23.6
**32**	**Lightner** Amy L et al; (2019)	40.5%	37.625	24.3
**33**	**Shim** Hang Hocj et al; (2018)	31.25%	33.5	23.6
**34**	**Aziz** Mohamed A Abd El et al; (2022)	43.4%	35	25

INFL vs Control

Study or Subgroup	Experimental Events	Experimental Total	Control Events	Control Total	Weight	Risk Ratio M-H, Random, 95% CI
Bregnbak 2012	6	20	7	51	6.8%	2.19 [0.84, 5.71]
Coquet-Reinier 2010	1	13	1	13	1.1%	1.00 [0.07, 14.34]
Kasparek 2012	7	48	2	48	3.2%	3.50 [0.77, 16.00]
Kim 2020	16	74	8	54	9.3%	1.46 [0.67, 3.16]
Kotze 2017	7	71	2	52	3.2%	2.56 [0.55, 11.84]
Krane 2013	11	142	18	376	10.1%	1.62 [0.78, 3.34]
Lightner 2017	12	126	20	172	11.0%	0.82 [0.42, 1.61]
Lightner 2018	9	107	9	105	7.7%	0.98 [0.41, 2.37]
Regueiro 2011	0	11	1	13	0.8%	0.39 [0.02, 8.69]
Shah 2021	19	113	142	505	17.2%	0.60 [0.39, 0.92]
Tang 2020	3	85	4	305	3.4%	2.69 [0.61, 11.79]
Waterman 2013	40	195	67	278	20.0%	0.85 [0.60, 1.20]
Yamada 2017	5	129	11	250	6.1%	0.88 [0.31, 2.48]
**Total (95% CI)**		**1134**		**2222**	**100.0%**	**1.08 [0.81, 1.45]**
Total events	136		292			

Heterogeneity: Tau² = 0.08; Chi² = 17.80, df = 12 (P = 0.12); I² = 33%
Test for overall effect: Z = 0.55 (P = 0.58)

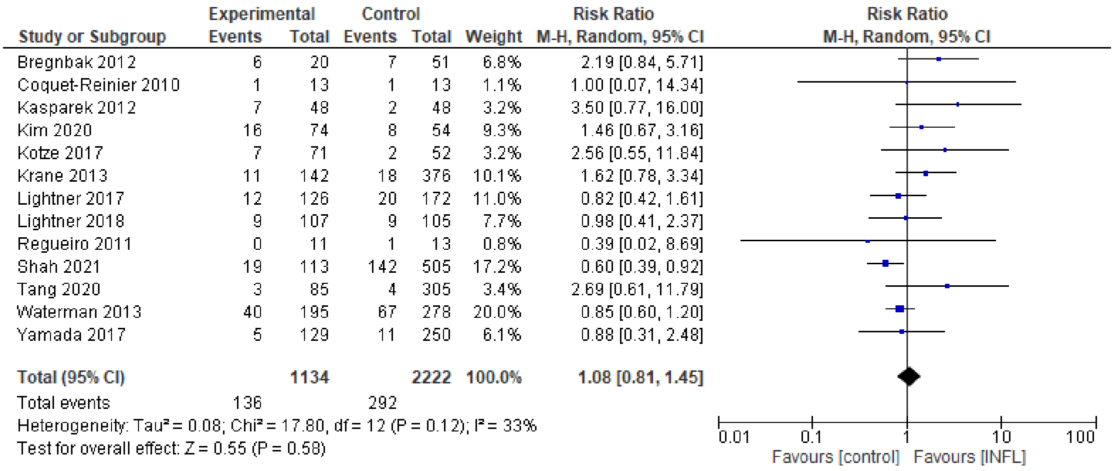

Figure S1.1: INFL vs CNTRL; postoperative ileus

Study or Subgroup	Experimental Events	Experimental Total	Control Events	Control Total	Weight	Risk Ratio M-H, Random, 95% CI
Bregnbak 2012	3	20	22	51	4.2%	0.35 [0.12, 1.03]
Coquet-Reinier 2010	1	13	0	13	1.0%	3.00 [0.13, 67.51]
Eshuis 2013	5	21	0	12	1.2%	6.50 [0.39, 108.26]
Gainsbury 2011	5	29	17	52	4.9%	0.53 [0.22, 1.28]
Kasparek 2012	12	48	12	48	5.7%	1.00 [0.50, 2.00]
Kotze 2017	15	71	19	52	6.2%	0.58 [0.33, 1.03]
Krane 2013	16	142	37	376	6.3%	1.15 [0.66, 1.99]
Kunitake 2008	6	101	32	312	5.1%	0.58 [0.25, 1.35]
Lightner 2017	11	126	22	172	5.7%	0.68 [0.34, 1.36]
Lightner 2018	10	107	22	172	5.7%	0.73 [0.36, 1.48]
Myrelid 2014	8	111	15	187	5.2%	0.90 [0.39, 2.05]
Norgard 2013	0	214	3	2079	1.1%	1.38 [0.07, 26.67]
Schluender 2007	3	17	11	134	3.9%	2.15 [0.67, 6.94]
Selvasekar 2007	18	47	38	254	6.6%	2.56 [1.61, 4.08]
Shah 2021	9	113	211	505	6.0%	0.19 [0.10, 0.36]
Tang 2020	49	85	80	305	7.3%	2.20 [1.69, 2.86]
Uchino 2013	2	22	46	562	3.4%	1.11 [0.29, 4.28]
Ward 2017	51	753	392	5472	7.2%	0.95 [0.71, 1.25]
Waterman 2013	44	195	43	278	6.9%	1.46 [1.00, 2.13]
Yamada 2017	19	129	34	250	6.4%	1.08 [0.64, 1.82]
**Total (95% CI)**		**2364**		**11286**	**100.0%**	**0.95 [0.68, 1.32]**
Total events	287		1056			

Heterogeneity: Tau² = 0.38; Chi² = 101.38, df = 19 (P < 0.00001); I² = 81%
Test for overall effect: Z = 0.33 (P = 0.74)

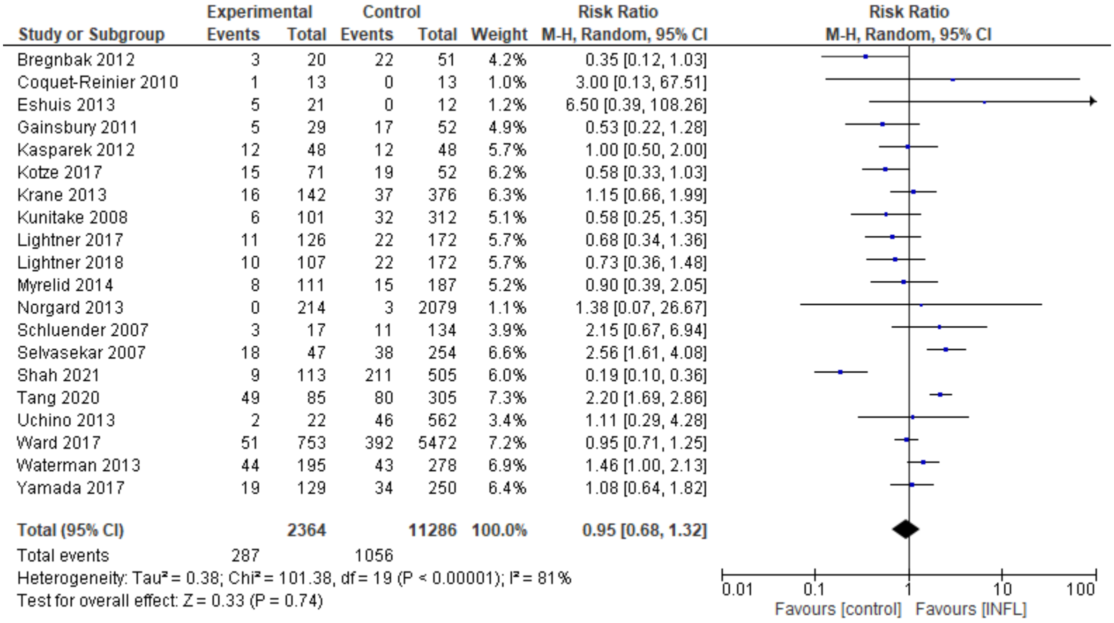

Figure S1.2: INFL vs CNTRL; SSIs

Study or Subgroup	Experimental Events	Experimental Total	Control Events	Control Total	Weight	Risk Ratio M-H, Random, 95% CI
Coquet-Reinier 2010	0	13	1	13	2.6%	0.33 [0.01, 7.50]
Gainsbury 2011	1	29	5	52	4.8%	0.36 [0.04, 2.92]
Kasparek 2012	2	48	6	48	7.3%	0.33 [0.07, 1.57]
Kotze 2017	8	71	4	52	10.1%	1.46 [0.47, 4.61]
Krane 2013	3	142	5	376	8.1%	1.59 [0.38, 6.56]
Kunitake 2008	3	101	9	312	9.0%	1.03 [0.28, 3.73]
Lightner 2017	4	126	1	172	4.6%	5.46 [0.62, 48.27]
Lightner 2018	0	107	3	105	2.8%	0.14 [0.01, 2.68]
Norgard 2013	8	214	181	2079	14.2%	0.43 [0.21, 0.86]
Selvasekar 2007	4	47	5	254	9.1%	4.32 [1.21, 15.51]
Shah 2021	1	113	20	505	5.2%	0.22 [0.03, 1.65]
Tang 2020	0	85	1	305	2.5%	1.19 [0.05, 28.85]
Waterman 2013	5	195	15	278	11.4%	0.48 [0.18, 1.29]
Yamada 2017	4	129	4	250	8.4%	1.94 [0.49, 7.62]
**Total (95% CI)**		**1420**		**4801**	**100.0%**	**0.86 [0.50, 1.48]**
Total events	43		260			

Heterogeneity: Tau² = 0.40; Chi² = 23.11, df = 13 (P = 0.04); I² = 44%
Test for overall effect: Z = 0.54 (P = 0.59)

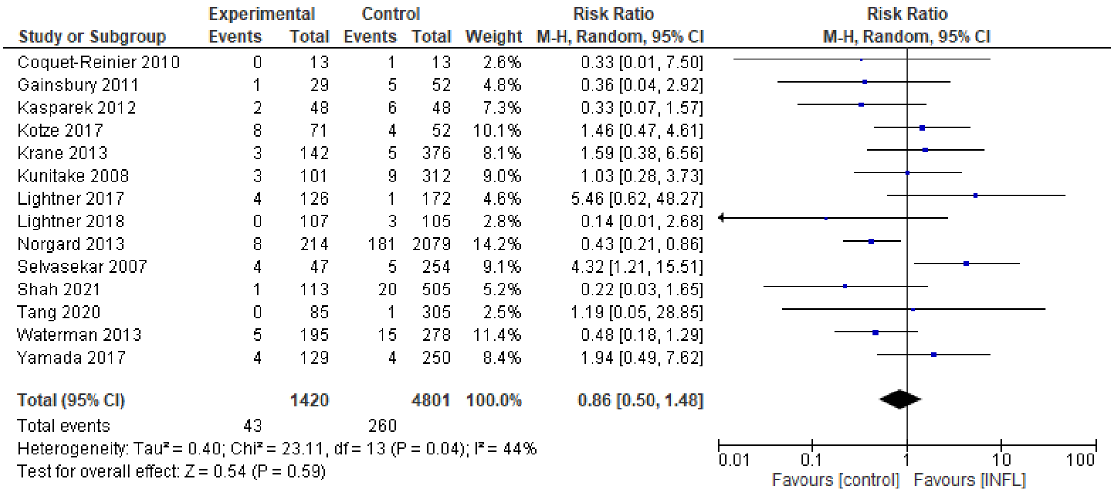

Figure S1.3: INFL vs CNTRL; anastomotic leakage

<u>VDLZ vs Control</u>

Study or Subgroup	Experimental Events	Experimental Total	Control Events	Control Total	Weight	Risk Ratio M-H, Random, 95% CI
Kim 2020	11	25	8	54	15.2%	2.97 [1.36, 6.46]
Lightner 2017	9	94	20	172	15.8%	0.82 [0.39, 1.74]
Lightner 2018	21	100	9	105	16.1%	2.45 [1.18, 5.09]
Novello 2020	28	95	18	95	21.1%	1.56 [0.93, 2.61]
Shah 2021	11	32	142	505	21.6%	1.22 [0.74, 2.01]
Yamada 2017	4	64	11	89	10.1%	0.51 [0.17, 1.52]
**Total (95% CI)**		**410**		**1020**	**100.0%**	**1.41 [0.92, 2.17]**
Total events	84		208			

Heterogeneity: Tau² = 0.15; Chi² = 11.52, df = 5 (P = 0.04); I² = 57%
Test for overall effect: Z = 1.59 (P = 0.11)

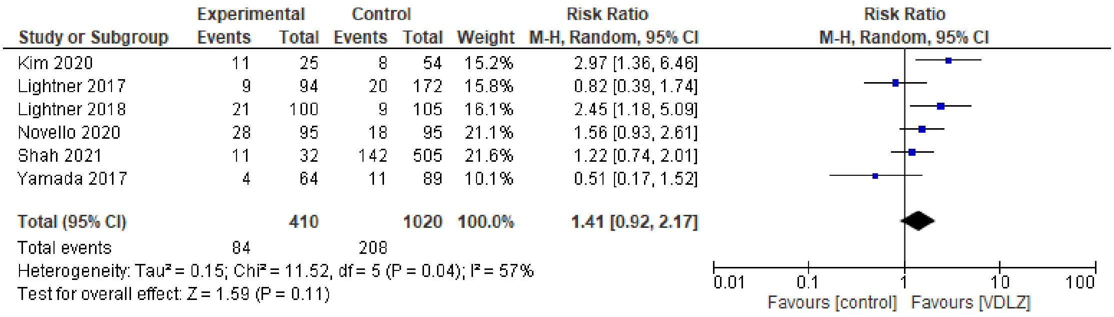

Figure S2.1: VDLZ vs CNTRL; postoperative ileus

Study or Subgroup	Experimental Events	Experimental Total	Control Events	Control Total	Weight	Risk Ratio M-H, Random, 95% CI
Kim 2020	2	25	9	54	9.4%	0.48 [0.11, 2.06]
Kotze 2017	6	34	1	36	5.9%	6.35 [0.81, 50.07]
Lightner 2017	33	94	22	172	20.1%	2.74 [1.70, 4.42]
Lightner 2018	23	100	10	105	17.5%	2.42 [1.21, 4.82]
Novello 2020	7	95	13	95	15.2%	0.54 [0.22, 1.29]
Shah 2021	20	32	211	505	22.0%	1.50 [1.12, 1.99]
Yamada 2017	2	64	34	250	9.9%	0.23 [0.06, 0.93]
**Total (95% CI)**		**444**		**1217**	**100.0%**	**1.28 [0.72, 2.28]**
Total events	93		300			

Heterogeneity: Tau² = 0.37; Chi² = 24.66, df = 6 (P = 0.0004); I² = 76%
Test for overall effect: Z = 0.83 (P = 0.40)

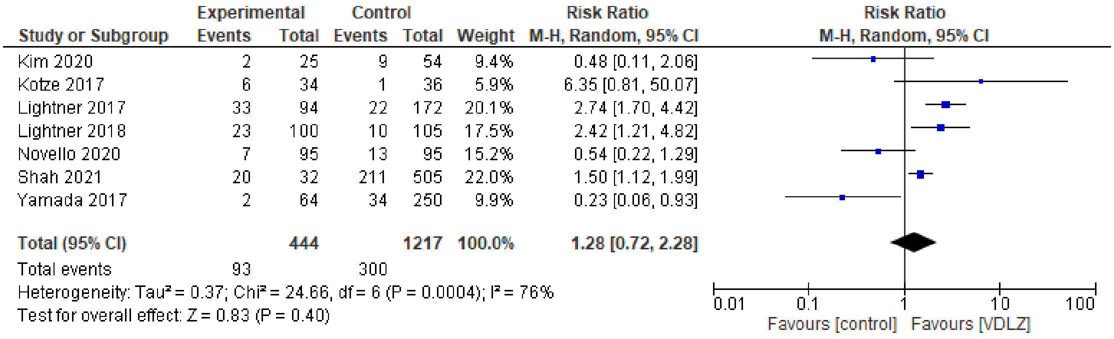

Figure S2.2: VDLZ vs CNTRL; SSIs

Study or Subgroup	Experimental Events	Experimental Total	Control Events	Control Total	Weight	Risk Ratio M-H, Random, 95% CI
Lightner 2017	2	94	1	72	12.8%	1.53 [0.14, 16.56]
Lightner 2018	1	100	3	105	14.4%	0.35 [0.04, 3.31]
Novello 2020	4	95	6	95	47.9%	0.67 [0.19, 2.29]
Shah 2021	0	32	20	505	9.4%	0.37 [0.02, 6.05]
Yamada 2017	1	64	4	250	15.4%	0.98 [0.11, 8.59]
**Total (95% CI)**		**385**		**1027**	**100.0%**	**0.68 [0.29, 1.59]**
Total events	8		34			

Heterogeneity: Tau² = 0.00; Chi² = 1.07, df = 4 (P = 0.90); I² = 0%
Test for overall effect: Z = 0.89 (P = 0.37)

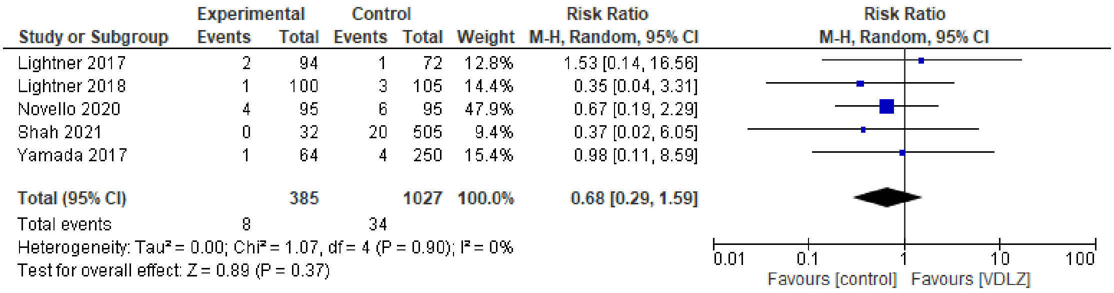

Figure S2.3: VDLZ vs CNTRL; SSIs

USTK vs Control

Study or Subgroup	USTK Events	USTK Total	Control Events	Control Total	Weight	Risk Ratio M-H, Random, 95% CI
El Aziz et al 2022	8	52	12	90	58.5%	1.15 [0.50, 2.64]
Shah et al 2021	2	21	142	505	41.5%	0.34 [0.09, 1.28]
**Total (95% CI)**		**73**		**595**	**100.0%**	**0.69 [0.20, 2.39]**
Total events	10		154			

Heterogeneity: Tau² = 0.50; Chi² = 2.57, df = 1 (P = 0.11); I² = 61%
Test for overall effect: Z = 0.58 (P = 0.56)

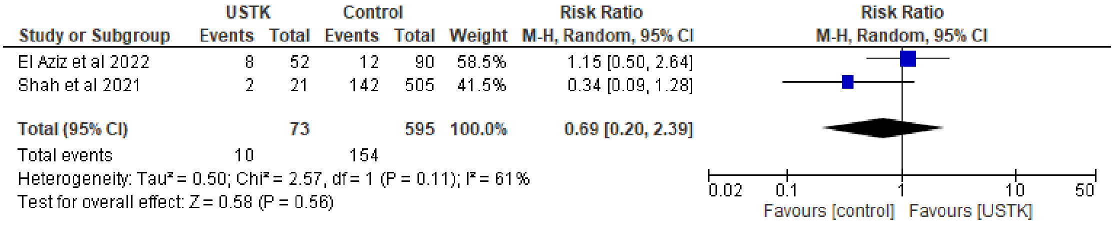

Figure S3.1: USTK vs Control; postoperative ileus

Study or Subgroup	USTK Events	USTK Total	Control Events	Control Total	Weight	Risk Ratio M-H, Random, 95% CI
El Aziz et al 2022	4	50	20	90	51.8%	0.36 [0.13, 0.99]
Shah et al 2021	3	21	211	505	48.2%	0.34 [0.12, 0.98]
**Total (95% CI)**		**71**		**595**	**100.0%**	**0.35 [0.17, 0.73]**
Total events	7		231			

Heterogeneity: Tau² = 0.00; Chi² = 0.00, df = 1 (P = 0.94); I² = 0%
Test for overall effect: Z = 2.81 (P = 0.005)

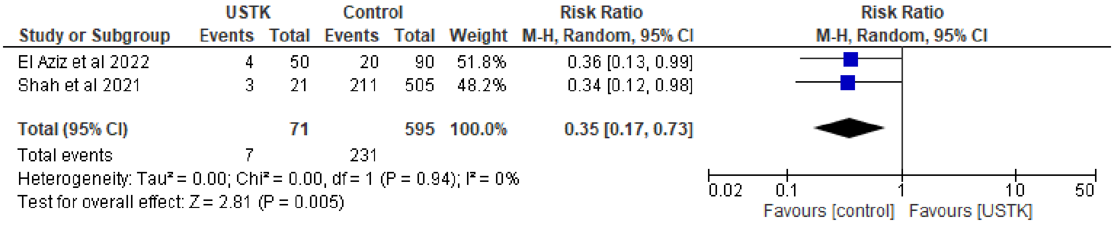

Figure S3.2: USTK vs Control; SSIs

Study or Subgroup	USTK Events	USTK Total	Control Events	Control Total	Weight	Risk Ratio M-H, Random, 95% CI
El Aziz et al 2022	21	52	0	90	50.0%	73.83 [4.57, 1194.03]
Shah et al 2021	0	21	20	505	50.0%	0.56 [0.04, 8.98]
**Total (95% CI)**		**73**		**595**	**100.0%**	**6.43 [0.04, 958.68]**
Total events	21		20			

Heterogeneity: Tau² = 11.03; Chi² = 6.49, df = 1 (P = 0.01); I² = 85%
Test for overall effect: Z = 0.73 (P = 0.47)

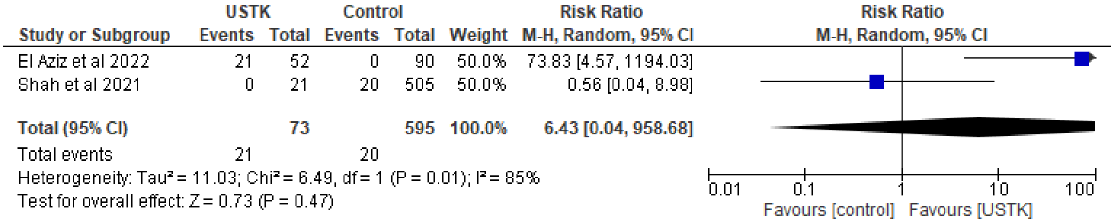

Figure S3.3: USTK vs Control; anastomotic leakage

USTK vs INFL

Study or Subgroup	USTK Events	USTK Total	INFL Events	INFL Total	Weight	Risk Ratio M-H, Random, 95% CI
El Aziz et al 2022	8	52	19	113	61.9%	0.91 [0.43, 1.95]
Shah et al 2021	2	21	58	257	19.9%	0.42 [0.11, 1.61]
Shim et al 2018	3	20	4	40	18.2%	1.50 [0.37, 6.07]
**Total (95% CI)**		**93**		**410**	**100.0%**	**0.86 [0.47, 1.56]**
Total events	13		81			

Heterogeneity: Tau² = 0.00; Chi² = 1.78, df = 2 (P = 0.41); I² = 0%
Test for overall effect: Z = 0.50 (P = 0.62)

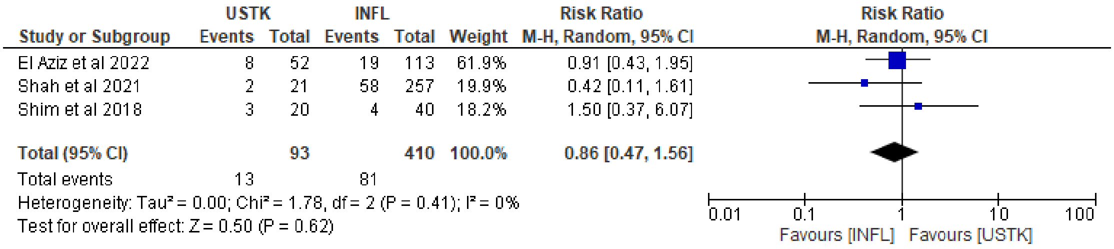

Figure S4.1: USTK vs INFL; postoperative ileus

Study or Subgroup	USTK Events	USTK Total	INFL Events	INFL Total	Weight	Risk Ratio M-H, Random, 95% CI
El Aziz et al 2022	4	52	9	113	39.3%	0.97 [0.31, 2.99]
Shah et al 2021	3	21	159	257	42.0%	0.23 [0.08, 0.66]
Shim et al 2018	1	20	6	40	18.7%	0.33 [0.04, 2.58]
**Total (95% CI)**		**93**		**410**	**100.0%**	**0.43 [0.16, 1.20]**
Total events	8		174			

Heterogeneity: Tau² = 0.35; Chi² = 3.57, df = 2 (P = 0.17); I² = 44%
Test for overall effect: Z = 1.61 (P = 0.11)

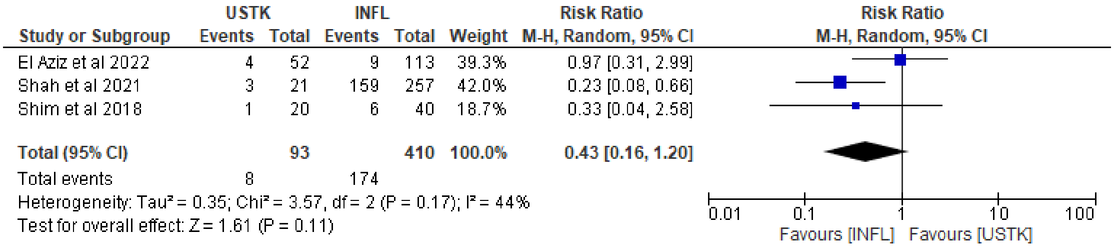

Figure S4.2: USTK vs INFL; SSIs

Study or Subgroup	USTK Events	USTK Total	INFL Events	INFL Total	Weight	Risk Ratio M-H, Random, 95% CI
El Aziz et al 2022	2	52	1	113	41.5%	4.35 [0.40, 46.86]
Shah et al 2021	0	21	6	257	29.9%	0.90 [0.05, 15.49]
Shim et al 2018	0	20	3	40	28.5%	0.28 [0.02, 5.15]
**Total (95% CI)**		**93**		**410**	**100.0%**	**1.24 [0.25, 6.21]**
Total events	2		10			

Heterogeneity: Tau² = 0.16; Chi² = 2.16, df = 2 (P = 0.34); I² = 8%
Test for overall effect: Z = 0.26 (P = 0.79)

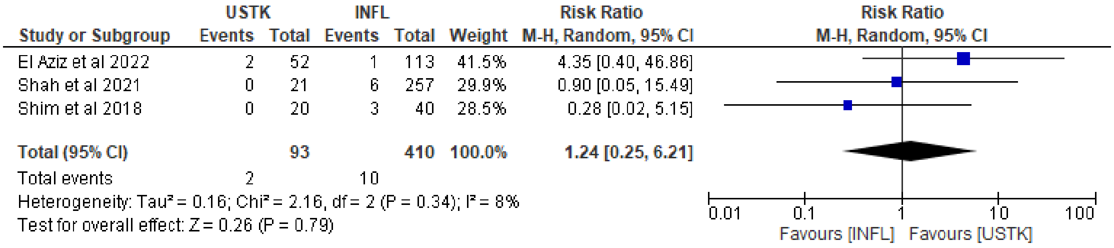

Figure S4.3: USTK vs INFL; anastomotic leakage

USTK vs VDLZ

Study or Subgroup	USTK Events	USTK Total	VDLZ Events	VDLZ Total	Weight	Risk Ratio M-H, Random, 95% CI
El Aziz et al 2022	8	52	1	19	45.2%	2.92 [0.39, 21.85]
Shah et al 2021	2	21	11	32	54.8%	0.28 [0.07, 1.13]
**Total (95% CI)**		**73**		**51**	**100.0%**	**0.80 [0.08, 8.06]**
Total events	10		12			

Heterogeneity: Tau² = 2.01; Chi² = 3.57, df = 1 (P = 0.06); I² = 72%
Test for overall effect: Z = 0.19 (P = 0.85)

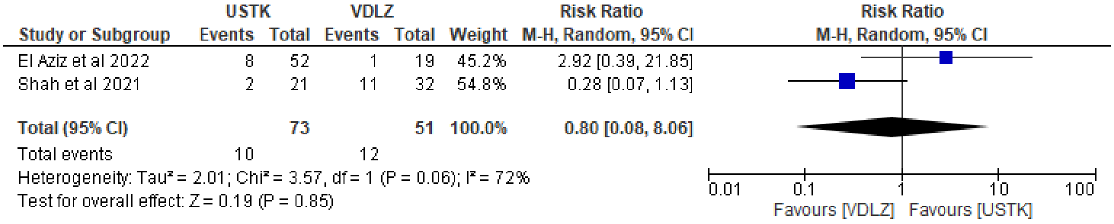

Figure S5.1: USTK vs VDLZ; postoperative ileus

Study or Subgroup	USTK Events	USTK Total	VDLZ Events	VDLZ Total	Weight	Risk Ratio M-H, Random, 95% CI
El Aziz et al 2022	4	52	1	19	28.7%	1.46 [0.17, 12.27]
Novello et al 2018	7	8	11	40	36.6%	3.18 [1.80, 5.61]
Shah et al 2021	3	21	20	32	34.7%	0.23 [0.08, 0.67]
**Total (95% CI)**		**81**		**91**	**100.0%**	**1.02 [0.10, 10.87]**
Total events	14		32			

Heterogeneity: Tau² = 3.89; Chi² = 27.27, df = 2 (P < 0.00001); I² = 93%
Test for overall effect: Z = 0.02 (P = 0.99)

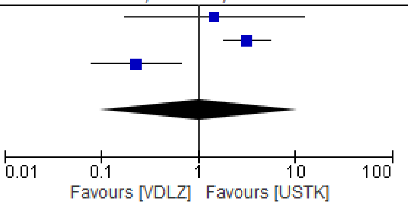

Figure S5.2: USTK vs VDLZ; SSIs

INFL vs VDLZ

Study or Subgroup	Experimental Events	Experimental Total	Control Events	Control Total	Weight	Risk Ratio M-H, Random, 95% CI
Kim 2020	11	25	13	94	29.1%	3.18 [1.63, 6.22]
Lightner 2017	7	88	5	62	10.8%	0.99 [0.33, 2.97]
Lightner 2018	21	100	9	108	24.4%	2.52 [1.21, 5.24]
Poylin 2022	12	38	13	94	27.7%	2.28 [1.15, 4.54]
Yamada 2017	4	64	5	129	8.0%	1.61 [0.45, 5.80]
**Total (95% CI)**		**315**		**487**	**100.0%**	**2.29 [1.59, 3.29]**
Total events	55		45			

Heterogeneity: Tau² = 0.00; Chi² = 3.58, df = 4 (P = 0.47); I² = 0%
Test for overall effect: Z = 4.48 (P < 0.00001)

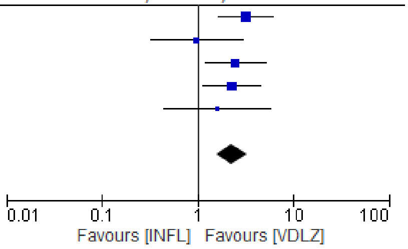

Figure S6.1: INFL vs VDLZ; postoperative ileus

Study or Subgroup	Experimental Events	Experimental Total	Control Events	Control Total	Weight	Risk Ratio M-H, Random, 95% CI
Ferrante 2017	4	34	9	60	12.4%	0.78 [0.26, 2.36]
Kim 2020	2	25	7	74	8.1%	0.85 [0.19, 3.81]
Lightner 2017	19	88	9	62	18.9%	1.49 [0.72, 3.07]
Lightner 2018	23	100	10	107	19.6%	2.46 [1.23, 4.91]
Park 2018	14	92	11	94	18.7%	1.30 [0.62, 2.71]
Poylin 2022	4	38	19	94	13.7%	0.52 [0.19, 1.43]
Yamada 2017	2	64	19	192	8.7%	0.32 [0.08, 1.32]
**Total (95% CI)**		**441**		**683**	**100.0%**	**1.07 [0.65, 1.76]**
Total events	68		84			

Heterogeneity: Tau² = 0.20; Chi² = 11.41, df = 6 (P = 0.08); I² = 47%
Test for overall effect: Z = 0.27 (P = 0.79)

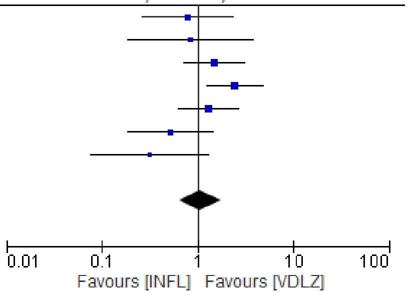

Figure S6.2: INFL vs VDLZ; SSIs

Study or Subgroup	Experimental Events	Experimental Total	Control Events	Control Total	Weight	Risk Ratio M-H, Random, 95% CI
Ferrante 2017	1	34	2	60	29.9%	0.88 [0.08, 9.38]
Lightner 2017	0	88	0	62		Not estimable
Lightner 2018	1	100	0	107	16.4%	3.21 [0.13, 77.84]
Poylin 2022	0	38	2	94	18.4%	0.49 [0.02, 9.92]
Yamada 2017	1	64	4	129	35.4%	0.50 [0.06, 4.42]
**Total (95% CI)**		**324**		**452**	**100.0%**	**0.80 [0.22, 2.92]**
Total events	3		8			

Heterogeneity: Tau² = 0.00; Chi² = 1.01, df = 3 (P = 0.80); I² = 0%
Test for overall effect: Z = 0.34 (P = 0.74)

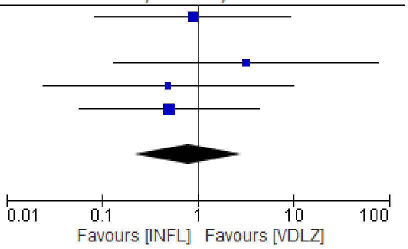

Figure S6.3: INFL vs VDLZ; anastomotic leakage

Suppl. Figure S8: Funnel Plots

Figure S8.1: INFL vs control - total surgical complications

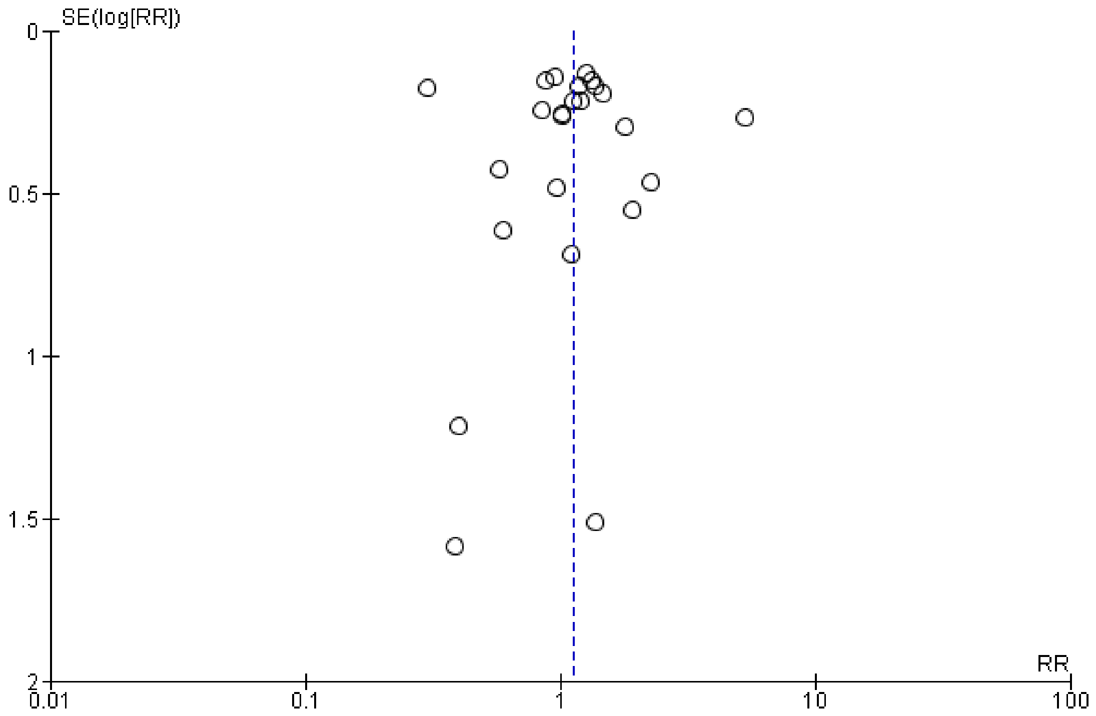

Figure S8.2: infl vs control - ileus

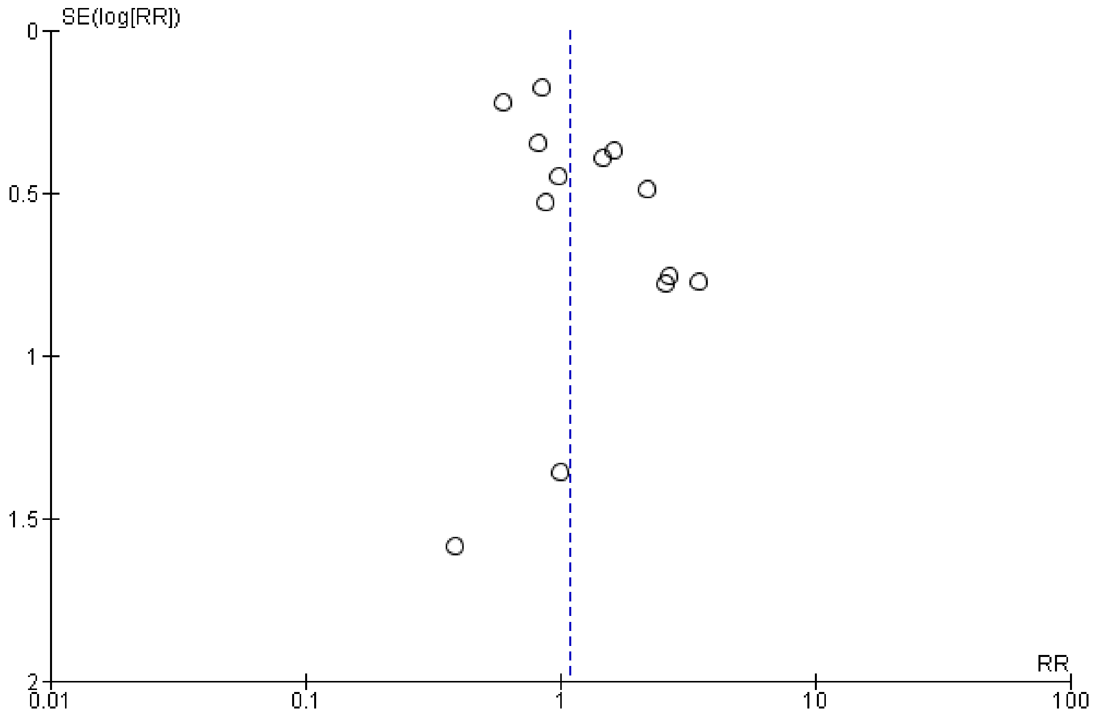

Figure S8.3: infl vs control – ssis

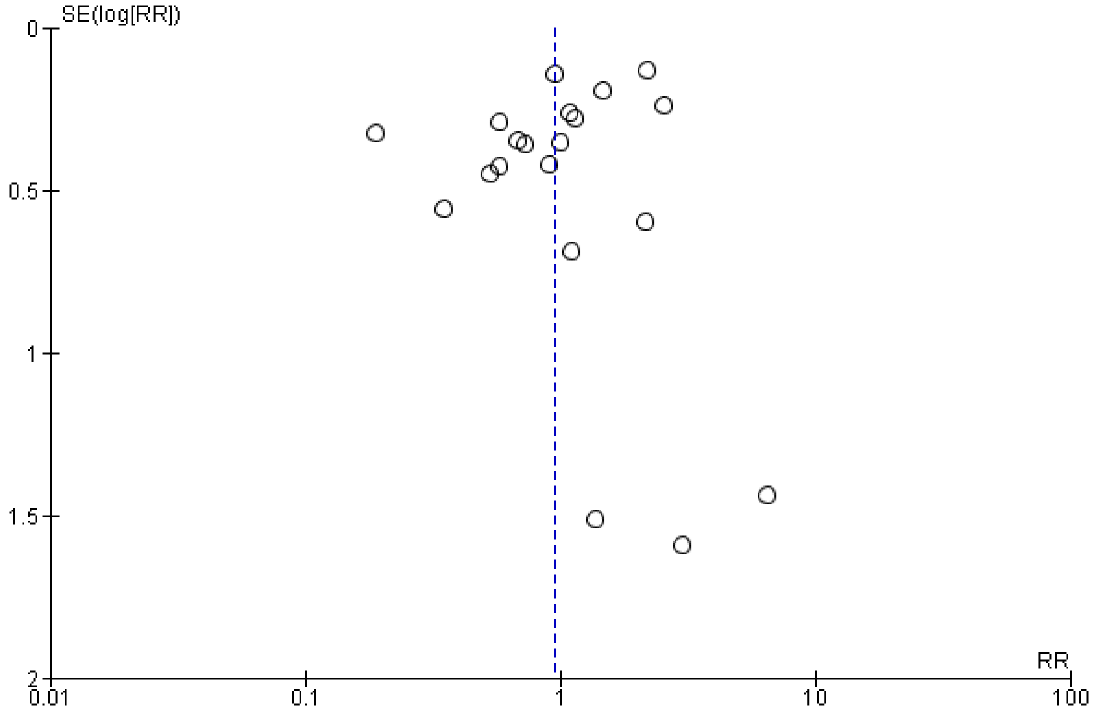

Figure S8.4: infl vs control - leak

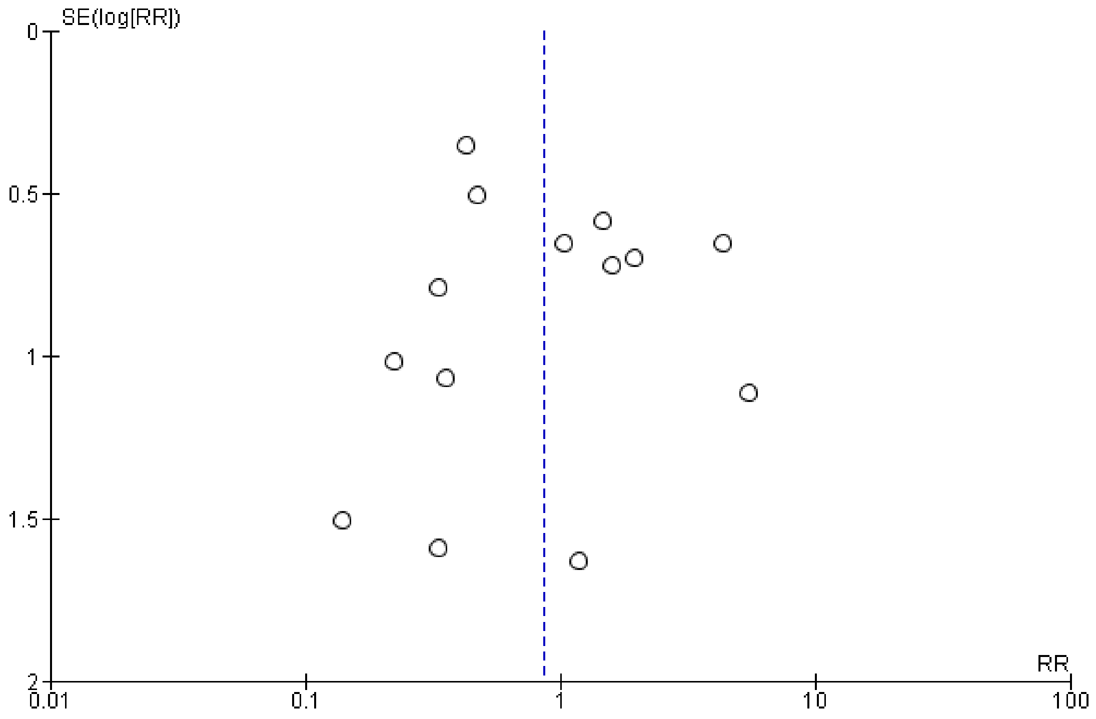

Supplementary Figure S9: Sensitivity and subgroup analysis' forest plots between INFL vs CNTRL

Figure S9.1: *Total Complications*

Study or Subgroup	Experimental Events	Experimental Total	Control Events	Control Total	Weight	Risk Ratio M-H, Random, 95% CI
**1.1.1 Crohn's disease**						
Coquet-Reinier 2010	3	13	5	13	2.3%	0.60 [0.18, 2.01]
El-Aziz 2022	1	113	2	90	0.8%	0.40 [0.04, 4.32]
Kasparek 2012	33	48	24	48	5.7%	1.38 [0.98, 1.93]
Kotze 2017	23	71	20	52	5.1%	0.84 [0.52, 1.36]
Myrelid 2014	38	111	54	187	5.7%	1.19 [0.84, 1.67]
Norgard 2013	0	214	3	2079	0.6%	1.38 [0.07, 26.67]
Regueiro 2011	0	11	1	13	0.5%	0.39 [0.02, 8.69]
Shah 2021	25	113	373	505	5.7%	0.30 [0.21, 0.42]
Tang 2020	35	85	94	305	5.8%	1.34 [0.99, 1.81]
**Subtotal (95% CI)**		**779**		**3292**	**32.2%**	**0.82 [0.48, 1.41]**
Total events	158		576			
Heterogeneity: Tau² = 0.42; Chi² = 61.63, df = 8 (P < 0.00001); I² = 87%						
Test for overall effect: Z = 0.71 (P = 0.48)						
**1.1.2 Ulcerative colitis**						
Bregnbak 2012	10	20	25	51	4.9%	1.02 [0.61, 1.72]
Eshuis 2013	10	21	3	12	2.7%	1.90 [0.65, 5.60]
Gainsbury 2011	13	29	23	52	5.0%	1.01 [0.61, 1.68]
Kim 2020	33	74	20	54	5.3%	1.20 [0.78, 1.85]
Schluender 2007	8	17	35	134	4.6%	1.80 [1.01, 3.21]
Selvasekar 2007	29	47	124	254	6.0%	1.26 [0.98, 1.64]
Uchino 2013	2	22	46	562	2.0%	1.11 [0.29, 4.28]
Ward 2017	51	753	392	5472	5.9%	0.95 [0.71, 1.25]
Zittan 2016	26	196	66	562	5.3%	1.13 [0.74, 1.73]
**Subtotal (95% CI)**		**1179**		**7153**	**41.8%**	**1.15 [1.00, 1.32]**
Total events	182		734			
Heterogeneity: Tau² = 0.00; Chi² = 6.30, df = 8 (P = 0.61); I² = 0%						
Test for overall effect: Z = 1.92 (P = 0.05)						
**1.1.3 Mixed population**						
El-Hussuna 2018	5	18	8	28	3.1%	0.97 [0.38, 2.51]
Krane 2013	36	142	18	376	4.8%	5.30 [3.11, 9.01]
Kunitake 2008	6	101	32	312	3.5%	0.58 [0.25, 1.35]
Lightner 2019	16	197	6	167	3.2%	2.26 [0.91, 5.65]
Waterman 2013	44	195	43	278	5.5%	1.46 [1.00, 2.13]
Yamada 2017	40	129	89	250	5.8%	0.87 [0.64, 1.18]
**Subtotal (95% CI)**		**782**		**1411**	**26.0%**	**1.45 [0.78, 2.73]**
Total events	147		196			
Heterogeneity: Tau² = 0.50; Chi² = 39.06, df = 5 (P < 0.00001); I² = 87%						
Test for overall effect: Z = 1.17 (P = 0.24)						
**Total (95% CI)**		**2740**		**11856**	**100.0%**	**1.13 [0.90, 1.42]**
Total events	487		1506			
Heterogeneity: Tau² = 0.21; Chi² = 111.78, df = 23 (P < 0.00001); I² = 79%						
Test for overall effect: Z = 1.02 (P = 0.31)						
Test for subgroup differences: Chi² = 2.00, df = 2 (P = 0.37), I² = 0.2%						

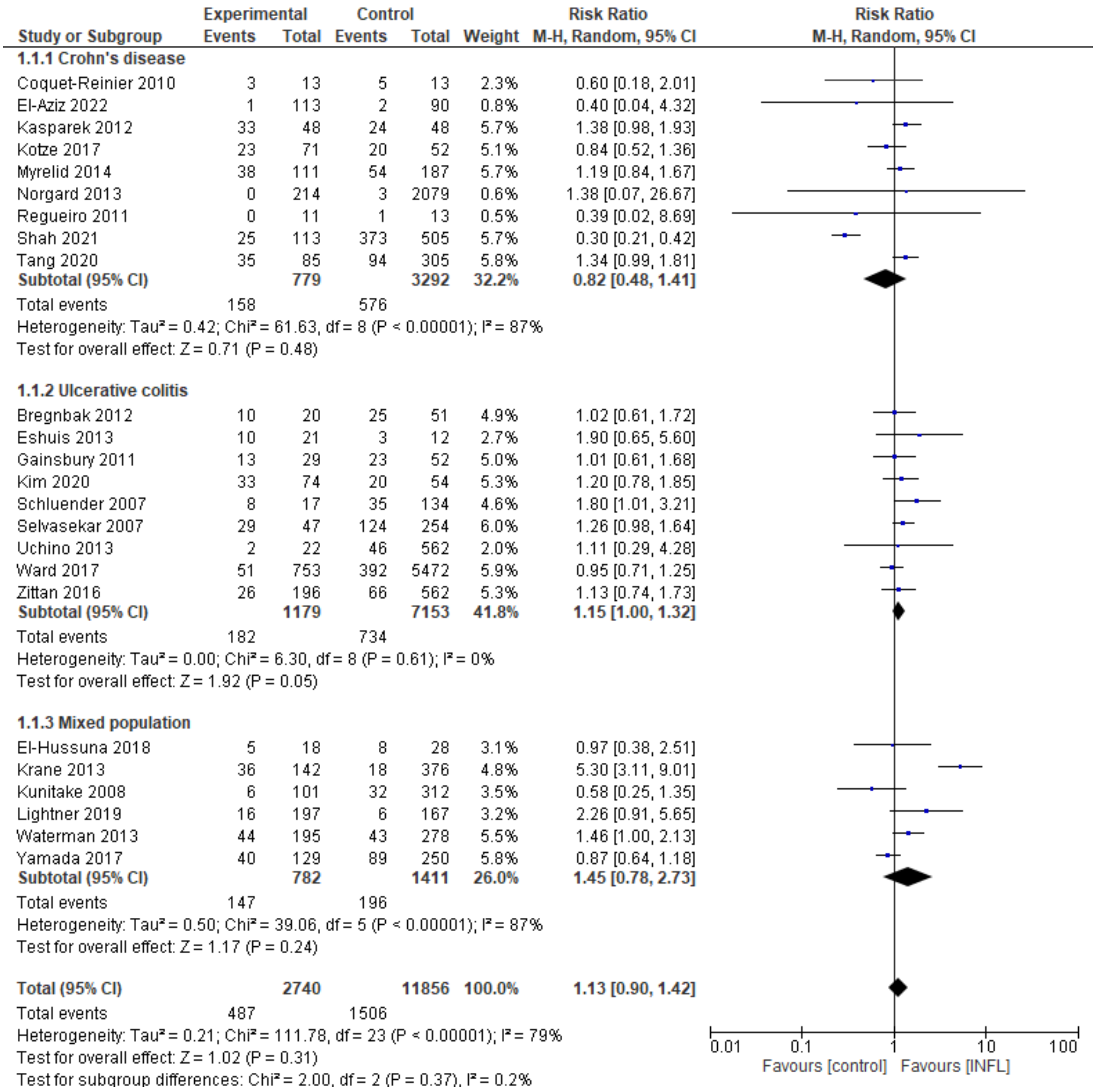

Figure S9.2: postoperative ileus

Study or Subgroup	Experimental Events	Experimental Total	Control Events	Control Total	Weight	Risk Ratio M-H, Random, 95% CI
**1.2.1 Crohn's disease**						
Kasparek 2012	7	48	2	48	3.2%	3.50 [0.77, 16.00]
Kotze 2017	7	71	2	52	3.2%	2.56 [0.55, 11.84]
Lightner 2018	9	107	9	105	7.7%	0.98 [0.41, 2.37]
Regueiro 2011	0	11	1	13	0.8%	0.39 [0.02, 8.69]
Shah 2021	19	113	142	505	17.2%	0.60 [0.39, 0.92]
Tang 2020	3	85	4	305	3.4%	2.69 [0.61, 11.79]
**Subtotal (95% CI)**		**435**		**1028**	**35.5%**	**1.24 [0.62, 2.49]**
Total events	45		160			
Heterogeneity: Tau² = 0.34; Chi² = 10.54, df = 5 (P = 0.06); I² = 53%						
Test for overall effect: Z = 0.61 (P = 0.54)						
**1.2.2 Ulcerative colitis**						
Bregnbak 2012	6	20	7	51	6.8%	2.19 [0.84, 5.71]
Coquet-Reinier 2010	1	13	1	13	1.1%	1.00 [0.07, 14.34]
Kim 2020	16	74	8	54	9.3%	1.46 [0.67, 3.16]
Lightner 2017	12	126	20	172	11.0%	0.82 [0.42, 1.61]
**Subtotal (95% CI)**		**233**		**290**	**28.3%**	**1.23 [0.79, 1.92]**
Total events	35		36			
Heterogeneity: Tau² = 0.00; Chi² = 2.99, df = 3 (P = 0.39); I² = 0%						
Test for overall effect: Z = 0.91 (P = 0.36)						
**1.2.3 Mixed population**						
Krane 2013	11	142	18	376	10.1%	1.62 [0.78, 3.34]
Waterman 2013	40	195	67	278	20.0%	0.85 [0.60, 1.20]
Yamada 2017	5	129	11	250	6.1%	0.88 [0.31, 2.48]
**Subtotal (95% CI)**		**466**		**904**	**36.2%**	**0.99 [0.68, 1.45]**
Total events	56		96			
Heterogeneity: Tau² = 0.03; Chi² = 2.48, df = 2 (P = 0.29); I² = 19%						
Test for overall effect: Z = 0.04 (P = 0.96)						
**Total (95% CI)**		**1134**		**2222**	**100.0%**	**1.08 [0.81, 1.45]**
Total events	136		292			
Heterogeneity: Tau² = 0.08; Chi² = 17.80, df = 12 (P = 0.12); I² = 33%						
Test for overall effect: Z = 0.55 (P = 0.58)						
Test for subgroup differences: Chi² = 0.64, df = 2 (P = 0.73), I² = 0%						

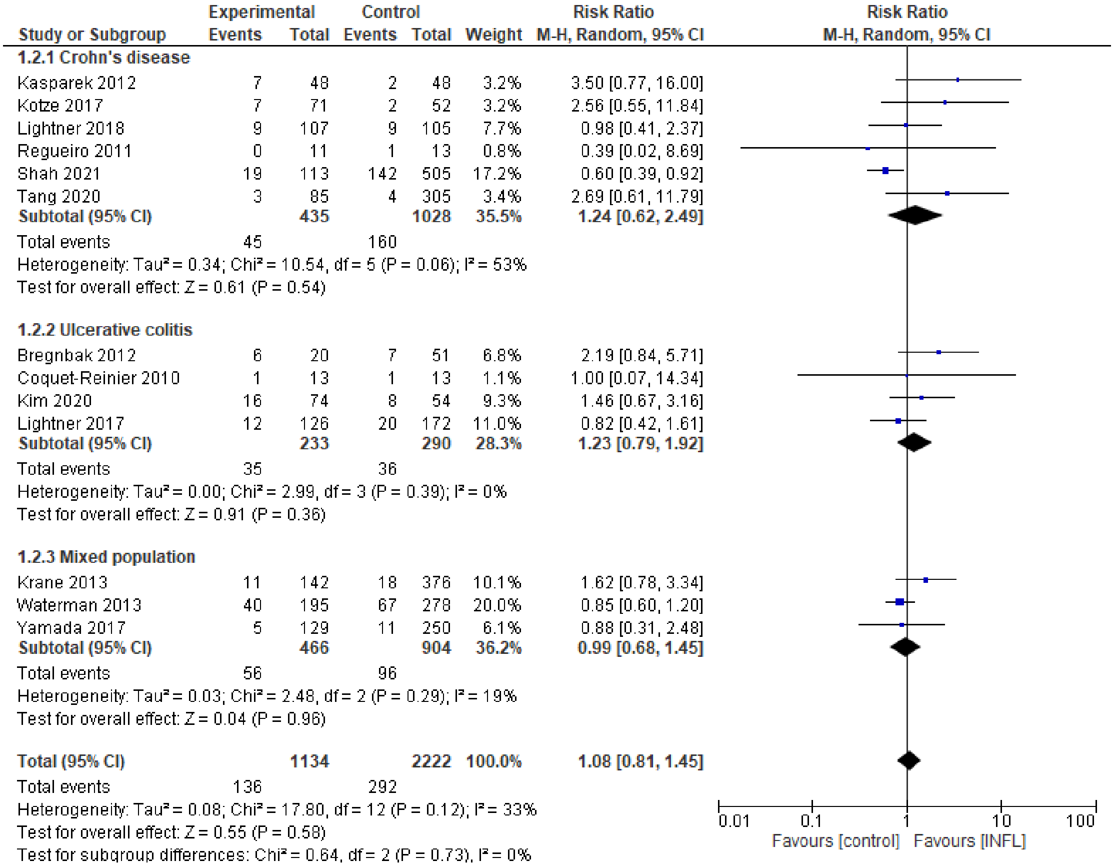

Figure S9.3: SSIs

Study or Subgroup	Experimental Events	Experimental Total	Control Events	Control Total	Weight	Risk Ratio M-H, Random, 95% CI
**1.3.1 Crohn's disease**						
Kasparek 2012	12	48	12	48	5.7%	1.00 [0.50, 2.00]
Kotze 2017	15	71	19	52	6.2%	0.58 [0.33, 1.03]
Lightner 2018	10	107	22	172	5.7%	0.73 [0.36, 1.48]
Myrelid 2014	8	111	15	187	5.2%	0.90 [0.39, 2.05]
Shah 2021	9	113	211	505	6.0%	0.19 [0.10, 0.36]
Tang 2020	49	85	80	305	7.3%	2.20 [1.69, 2.86]
Ward 2017	51	753	392	5472	7.2%	0.95 [0.71, 1.25]
**Subtotal (95% CI)**		**1288**		**6741**	**43.2%**	**0.78 [0.41, 1.47]**
Total events	154		751			
Heterogeneity: Tau² = 0.64; Chi² = 74.64, df = 6 (P < 0.00001); I² = 92%						
Test for overall effect: Z = 0.78 (P = 0.44)						
**1.3.2 Ulcerative colitis**						
Bregnbak 2012	3	20	22	51	4.2%	0.35 [0.12, 1.03]
Coquet-Reinier 2010	1	13	0	13	1.0%	3.00 [0.13, 67.51]
Eshuis 2013	5	21	0	12	1.2%	6.50 [0.39, 108.26]
Gainsbury 2011	5	29	17	52	4.9%	0.53 [0.22, 1.28]
Lightner 2017	11	126	22	172	5.7%	0.68 [0.34, 1.36]
Norgard 2013	0	214	3	2079	1.1%	1.38 [0.07, 26.67]
Schluender 2007	3	17	11	134	3.9%	2.15 [0.67, 6.94]
Selvasekar 2007	18	47	38	254	6.6%	2.56 [1.61, 4.08]
Uchino 2013	2	22	46	562	3.4%	1.11 [0.29, 4.28]
**Subtotal (95% CI)**		**509**		**3329**	**32.1%**	**1.13 [0.58, 2.19]**
Total events	48		159			
Heterogeneity: Tau² = 0.56; Chi² = 24.50, df = 8 (P = 0.002); I² = 67%						
Test for overall effect: Z = 0.35 (P = 0.73)						
**1.3.3 Mixed population**						
Krane 2013	16	142	37	376	6.3%	1.15 [0.66, 1.99]
Kunitake 2008	6	101	32	312	5.1%	0.58 [0.25, 1.35]
Waterman 2013	44	195	43	278	6.9%	1.46 [1.00, 2.13]
Yamada 2017	19	129	34	250	6.4%	1.08 [0.64, 1.82]
**Subtotal (95% CI)**		**567**		**1216**	**24.7%**	**1.14 [0.84, 1.56]**
Total events	85		146			
Heterogeneity: Tau² = 0.03; Chi² = 4.10, df = 3 (P = 0.25); I² = 27%						
Test for overall effect: Z = 0.85 (P = 0.40)						
**Total (95% CI)**		**2364**		**11286**	**100.0%**	**0.95 [0.68, 1.32]**
Total events	287		1056			
Heterogeneity: Tau² = 0.38; Chi² = 101.38, df = 19 (P < 0.00001); I² = 81%						
Test for overall effect: Z = 0.33 (P = 0.74)						
Test for subgroup differences: Chi² = 1.17, df = 2 (P = 0.56), I² = 0%						

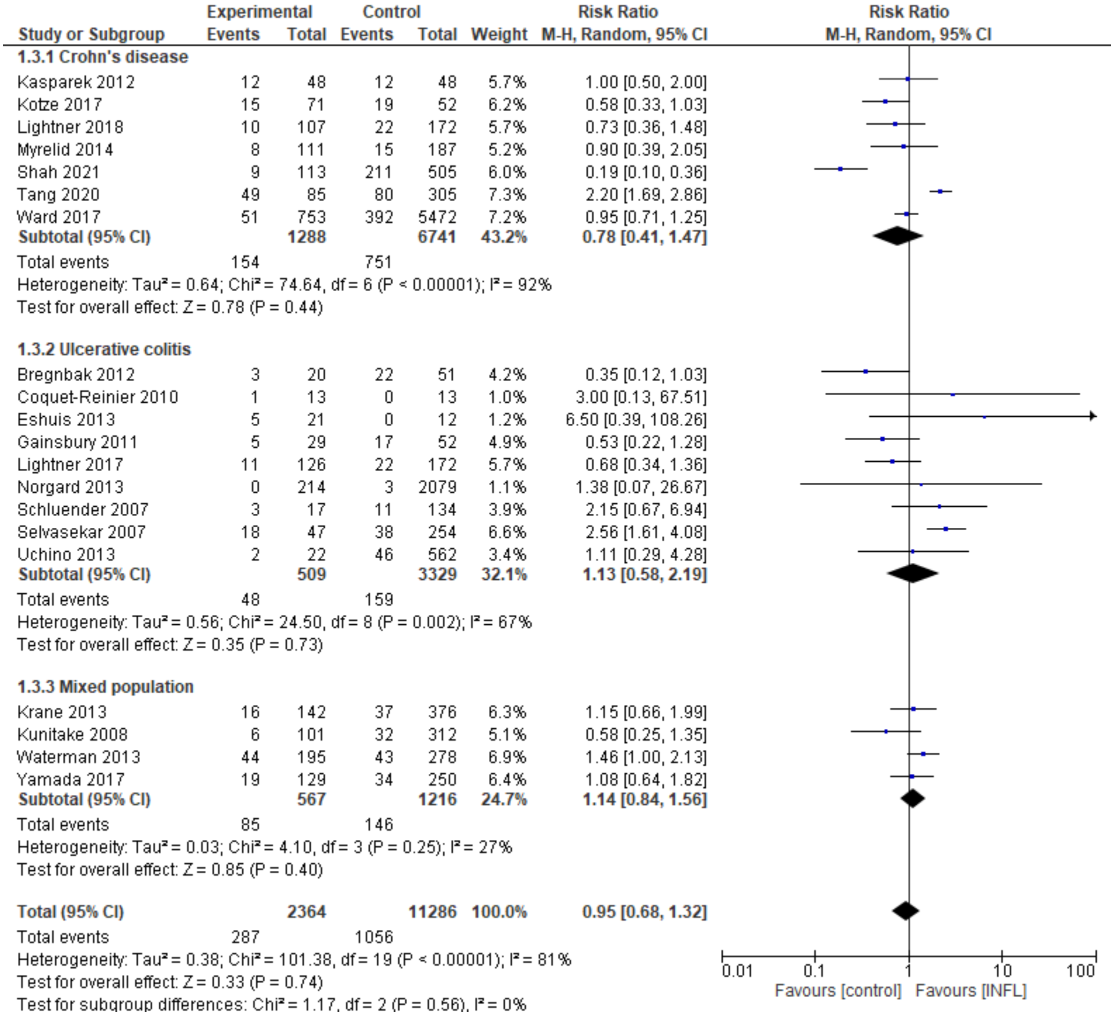

Figure S9.4: *Anastomotic Leakage*

Study or Subgroup	Experimental Events	Experimental Total	Control Events	Control Total	Weight	Risk Ratio M-H, Random, 95% CI
**1.4.1 Crohn's disease**						
Kasparek 2012	2	48	6	48	7.3%	0.33 [0.07, 1.57]
Kotze 2017	8	71	4	52	10.1%	1.46 [0.47, 4.61]
Lightner 2018	0	107	3	105	2.8%	0.14 [0.01, 2.68]
Norgard 2013	8	214	181	2079	14.2%	0.43 [0.21, 0.86]
Shah 2021	1	113	20	505	5.2%	0.22 [0.03, 1.65]
Tang 2020	0	85	1	305	2.5%	1.19 [0.05, 28.85]
**Subtotal (95% CI)**		**638**		**3094**	**42.1%**	**0.51 [0.28, 0.93]**
Total events	19		215			
Heterogeneity: Tau² = 0.06; Chi² = 5.54, df = 5 (P = 0.35); I² = 10%						
Test for overall effect: Z = 2.21 (P = 0.03)						
**1.4.2 Ulcerative colitis**						
Coquet-Reinier 2010	0	13	1	13	2.6%	0.33 [0.01, 7.50]
Gainsbury 2011	1	29	5	52	4.8%	0.36 [0.04, 2.92]
Lightner 2017	4	126	1	172	4.6%	5.46 [0.62, 48.27]
Selvasekar 2007	4	47	5	254	9.1%	4.32 [1.21, 15.51]
**Subtotal (95% CI)**		**215**		**491**	**21.1%**	**1.66 [0.38, 7.28]**
Total events	9		12			
Heterogeneity: Tau² = 1.15; Chi² = 6.25, df = 3 (P = 0.10); I² = 52%						
Test for overall effect: Z = 0.67 (P = 0.50)						
**1.4.3 Mixed population**						
Krane 2013	3	142	5	376	8.1%	1.59 [0.38, 6.56]
Kunitake 2008	3	101	9	312	9.0%	1.03 [0.28, 3.73]
Waterman 2013	5	195	15	278	11.4%	0.48 [0.18, 1.29]
Yamada 2017	4	129	4	250	8.4%	1.94 [0.49, 7.62]
**Subtotal (95% CI)**		**567**		**1216**	**36.9%**	**0.97 [0.50, 1.88]**
Total events	15		33			
Heterogeneity: Tau² = 0.06; Chi² = 3.44, df = 3 (P = 0.33); I² = 13%						
Test for overall effect: Z = 0.09 (P = 0.92)						
**Total (95% CI)**		**1420**		**4801**	**100.0%**	**0.86 [0.50, 1.48]**
Total events	43		260			
Heterogeneity: Tau² = 0.40; Chi² = 23.11, df = 13 (P = 0.04); I² = 44%						
Test for overall effect: Z = 0.54 (P = 0.59)						
Test for subgroup differences: Chi² = 3.25, df = 2 (P = 0.20), I² = 38.4%						

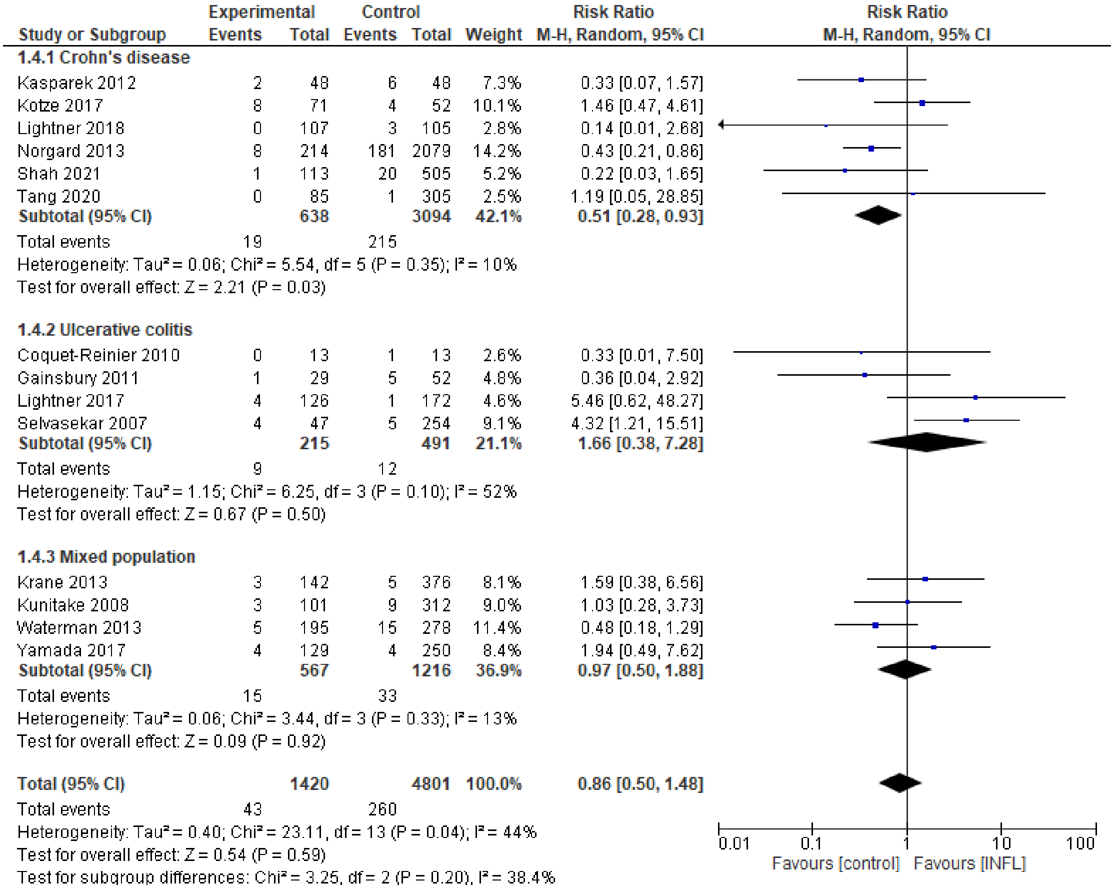

Suppl. Table S2: QUIPS Tool; Bias Assessment of including studies

Study	***Study Participation***	***Study Attrition***	***Prognostic Factor Measurement***	***Outcome Measurement***	***Study Confounding***	***Statistical Analysis and Reporting***
**Tang** Shasha et al; (2020)	low	high	low to moderate	low	low to moderate	low to moderate
El-**Hussuna** Alaa et al; (2018)	low to moderate	low to moderate	low	low to moderate	low to moderate	low to moderate
**Ward** S. T et al; (2017)	low to moderate	low	low to moderate	low	low	low to moderate
**Zittan** Eran et al; (2016)	low	low to moderate	low to moderate	low to moderate	low to moderate	low to moderate
p. **Myrelid** et al; (2014)	low to moderate	low	high	low to moderate	Low	low
**Uchino** Motoi et al; (2013)	low to moderate	low to moderate	low to moderate	low to moderate	low to moderate	low to moderate
**Uchino** Motoi et al; (2013)	low to moderate	low to moderate	low	low	high	low to moderate
**Krane** Mukta K et al; (2013)	low to moderate	low	low to moderate	low to moderate	low	low to moderate
**Waterman** Matti et al; (2013)	low to moderate	low to moderate	low to moderate	low	low to moderate	low
**Eshuis** Emma J et al; (2013)	Low	low to moderate	low to moderate	low to moderate	low to moderate	High
**Norgard** B M et al; (2013)	low to moderate	low to moderate	low to moderate	low	low to moderate	low to moderate
**Kasparek** M. S et al; (2012)	low to moderate	high	low	low to moderate	low to moderate	low
**Norgard** B M et al; (2012)	low	low to moderate	low to moderate	low to moderate	low	low to moderate
**Bregnbak** D. et al; (2012)	low	low to moderate	high	low to moderate	low	low
**Regueiro** Miguel et al; (2011)	low to moderate	low	low to moderate	low to moderate	low	
**Gainsbury** M. L et al; (2011)	low	low to moderate	low to moderate	low	low to moderate	high
**Coquet-Reinier** B. et al; (2010)	low to moderate	low to moderate	low to moderate	low	low	low to moderate
**Kunitake** Hiroko et al; (2008)	low to moderate	Low	low	high	low to moderate	low
**Schluender** Stefanie J et al; (2007)	low	low to moderate	low	low to moderate	low to moderate	low to moderate

Study	***Study Participation***	***Study Attrition***	***Prognostic Factor Measurement***	***Outcome Measurement***	***Study Confounding***	***Statistical Analysis and Reporting***
**Selvasekar** C. R et al (2007)	low to moderate	low to moderate	low to moderate	low	low to moderate	low
**Poylin** Vitaliy Y et al; (2022)	low	Low	Low	low to moderate	low to moderate	low to moderate
**Kim** Jeong Yeon et al; (2020)	low	low to moderate	low to moderate	low	low to moderate	low to moderate
**Novello** M et al; (2020)	low to moderate	low	low to moderate	low to moderate	Low	low to moderate
**Park** K T et al; (2018)	low to moderate	low to moderate	low to moderate	low to moderate	low to moderate	low to moderate
**Kotze** P.G. et al; (2018)	low to moderate	high	low	low to moderate	low to moderate	low to moderate
**Lightner** Amy L et al; (2018)	Low	low	low to moderate	moderate	low	low
**Lightner** A L et al; (2018)	low	low	low to moderate	low to moderate	low	low to moderate
**Lightner** Amy L et al; (2017)	low to moderate	low to moderate	low to moderate	low to moderate	low	low
**Ferrante** Marc et al; (2017)	low to moderate	low to moderate	low	low to moderate	low	low
**Yamada** Akihiro et al; (2017)	low to moderate	low	low to moderate	low to moderate	low to moderate	low
**Shah** Ravi S et al; (2021)	low	low to moderate	low to moderate	low to moderate	moderate	low to moderate
**Lightner** Amy L et al; (2019)	low to moderate	low to moderate	low	low	low to moderate	low to moderate
**Shim** Hang Hocj et al; (2018)	low to moderate	low to moderate	low	low to moderate	low to moderate	high
**Aziz** Mohamed A Abd El et al; (2022)	low	low to moderate	low to moderate	low to moderate	low	low to moderate
